# A Unique Result in Obstetrics

**Published:** 1887-12

**Authors:** W. J. Jolly

**Affiliations:** Waldo, Fla.


### A UNIQUE RESULT IN OBSTETRICS—THE PASSAGE OF LOWER SEGMENT OF COCCYX TEN DAYS AFTER LABOR PER ANUM.

*To the Atlanta Medical and Surgical Journal:*

I was called to Mrs. M., November 1st, 1887, primiparæ, aged 21 years, who was in labor. Nothing unusual occurred until the head was pressed against the coccyx, which did not yield. I applied the forceps and delivered her without any trouble and without any laceration of perineum. Immediately after delivery she suffered intense pain in the region of the coccyx, for which I gave an opiate and examined the bone. Found some displacement which I corrected, supposing it to be fractured. The opiate soon relieved the pain ; she did not suffer any more until the 9th day, except some tenderness in the region. She had some slight pain on that day. On the 10th she passed a bone per anum and sent it to me, stating that she thought she had passed a joint of her backbone. Upon examination I found it to be the lower segment of the coccyx. She has had no trouble since. As I have not seen a similar case on record, I send it to you for publication.

W. J. JOLLY, M. D.

*Waldo, Fla., Nov. 7, 1887.*

